# Diversity, expression, and structural modeling of sugar transporters in *Anisakis simplex* s. s. L3 and L4 larvae: an *in vitro* and *in silico* study

**DOI:** 10.3389/fcimb.2025.1621051

**Published:** 2025-08-20

**Authors:** Iwona Polak, Robert Stryiński, Łukasz Paukszto, Jan Paweł Jastrzębski, Iwona Bogacka, Elżbieta Łopieńska-Biernat

**Affiliations:** ^1^ Department of Biochemistry, Faculty of Biology and Biotechnology, University of Warmia and Mazury in Olsztyn, Olsztyn, Poland; ^2^ Department of Botany and Evolutionary Ecology, Faculty of Biology and Biotechnology, University of Warmia and Mazury in Olsztyn, Olsztyn, Poland; ^3^ Department of Plant Physiology, Genetics and Biotechnology, Faculty of Biology and Biotechnology, University of Warmia and Mazury in Olsztyn, Olsztyn, Poland; ^4^ Department of Animal Anatomy and Physiology, Faculty of Biology and Biotechnology, University of Warmia and Mazury in Olsztyn, Olsztyn, Poland

**Keywords:** *Anisakis*, GLUT transporters, facilitated glucose transporters, glucose, gene expression

## Abstract

**Introduction:**

Glucose transporter (GLUT) research in parasitic nematodes focuses on identifying and characterizing developmentally regulated isoforms, elucidating their regulatory and structural properties, and evaluating their potential as drug targets. While glucose transport mechanisms have been well characterized in the free-living nematode *Caenorhabditis elegans*, data on parasitic species remain limited. *Anisakis simplex* s. s., a parasitic nematode, relies on host-derived glucose to maintain energy metabolism. It is hypothesized that *A. simplex* s. s. utilizes specific glucose transporters to facilitate sugar uptake under varying nutritional conditions.

**Materials and methods:**

*In silico* analysis identified five putative facilitated glucose transporter genes (*fgt-1, fgt-2, fgt-3, fgt-5, fgt-9*) and one Sugars Will Eventually be Exported Transporter (*sweet-1*) gene. The FGTs were classified as members of the solute carrier family 2 (SLC2), while *sweet-1* belonged to the SWEET transporter family. Full-length cDNA sequences were obtained, and encoded proteins structurally characterized using bioinformatic modeling. Expression of transporter genes was assessed in *A. simplex* s. s. larvae at stages L3 and L4 cultured *in vitro* under different glucose concentrations and time points.

**Results:**

Structural and phylogenetic analyses revealed that *fgt-1* and *fgt-3* share high similarity with class I GLUTs found in nematodes and vertebrates. Gene expression profiling demonstrated differential regulation between larval stages. Most notably, FGT genes were stably expressed in L4 larvae, whereas in L3 larvae, gene activation was more variable and dependent on glucose concentration, showing a dynamic transcriptional response to nutrient levels. *Sweet-1* was expressed in both stages, but its regulation differed over time and with glucose availability. Glucose supplementation altered trehalose and glycogen levels, and trehalase activity varied across stages and treatments, indicating stage-specific metabolic adaptation.

**Discussion:**

The observed transcriptional and biochemical differences between L3 and L4 larvae suggest a shift in glucose uptake mechanisms, from transcuticular absorption in L3 to intestinal glucose uptake in L4 following intestine activation. FGT1 and FGT3 are proposed as key facilitators of glucose uptake, with roles varying across developmental stages. These findings indicate that glucose transporters are regulated in response to changing environmental conditions and may represent targets for rational anthelmintic drug design.

## 1 Introduction

Glucose is an essential fuel for most living organisms on Earth. As a polar and hydrophilic molecule, it cannot pass through hydrophobic cell membranes. The current state of knowledge has characterized several glucose transporters, including glucose transporters (GLUTs) belonging to the SLC2A gene family, sodium-glucose symporters (SGLTs), and SWEETs (Sugars Will Eventually be Exported Transporters) (Deng and Yan, 2016). GLUTs and SWEETs are uniporters that facilitate sugar transport along the sugar gradient (Chen et al., 2015). The large GLUT family consists of evolutionarily conserved facilitative glucose transporters involved in all critical steps of glucose and other hexose handling, including absorption, distribution, excretion, and recovery (Drew et al., 2021). Research on nematodes has predominantly focused on the free-living *C. elegans*, while studies on parasitic nematodes remain insufficient. Most developmental stages of gastrointestinal parasites occur under anaerobic conditions, where larvae derive most of their energy from saccharides. The FGT-1 (facilitated glucose transporter) of *C. elegans* plays a key role in glucose energy supply (Feng et al., 2013). The activity of FGT transport proteins was confirmed in the nematode intestine, as a GFP fusion protein of FGT-1 was observed exclusively on the basolateral membrane of digestive tract epithelia in *C. elegans*, but not in other tissues (Kitaoka et al., 2013) and recent data also showed that fluorescent glucose taken into the intestine of worms accumulates in the apical brush border (Saito et al., 2024). SWEET transporters play an important role in pathogenesis and stress response. In *C. elegans*, the SWEET homolog (*swt-1*) participates in glucose and trehalose transport and plays a crucial physiological role. RNAi-mediated inhibition of *swt-1* expression resulted in fewer offspring, changes in lifespan, and altered lipid content (Xuan et al., 2013; Jia et al., 2017).

The mechanisms of nutrient uptake in nematodes remain unclear. It is known that the composition of cuticle proteins changes during development (Iglesias et al., 2001; Łopieńska-Biernat et al., 2019a). The lack of anatomical channels, the thickness and complex structure of the cuticle, and the presence of a digestive tract seem to limit the uptake of substances between the external and internal environment in adult nematodes. However, the situation differs in larval forms with an incompletely developed intestine (Basyoni and Rizk, 2016). Specific gene expression is related to the functions and pathogenicity of different developmental stages in parasites (Schwarz et al., 2013). *A. simplex* s. s., a cosmopolitan parasitic nematode of marine organisms, which is characterized by a complex developmental cycle, requires glucose from the host environment to fulfill basic physiological functions such as energy metabolism. If glucose cannot be transported from the host environment, the parasite utilizes endogenous sources, as indicated by the specific metabolism of trehalose and glycogen in *A. simplex* larvae (Łopieńska-Biernat et al., 2019a, 2019b). These differences result from changes in diet and food intake methods. In L3 larvae of *A. simplex*, the intestinal lumen is shrunken, becoming functional only after the third molt in the L4 larvae (Grabda, 1976). Moreover, knowledge about the mechanisms of glucose absorption from the *A. simplex* intestinal lumen remains limited. Research on nematodes suggests that glucose is absorbed in the intestine, including against the concentration gradient. It likely assumes a key nutritional role after molting (Kitaoka et al., 2013). There is no conclusive evidence for the turnover of epicuticular materials between larval molts and in the adult stage (Howells, 1987). These phenomena are very interesting and require further directional research, especially since these processes may turn out to be a primary and perhaps universal mechanism.

Due to the increasing number of reported cases of anisakiasis, the study of this mechanism in both larval stages is particularly intriguing and could be universal for other gastrointestinal parasitic nematodes. Massive infestations by *A. simplex* L3 larvae have been observed in Baltic herring and cod (Mattiucci et al., 2016). The larvae of genus *Anisakis* are responsible for a disease known as anisakiasis in humans. The panel of experts identified *A. simplex* as a biohazardous organism (EFSA, 2010; Koutsoumanis et al., 2024). Anisakiasis is becoming a global problem in an era of widespread travel and rapid growth of international trade (Baird et al., 2014). The prevalence of fish infections caused by nematodes of the genus *Anisakis* is on the rise, and these parasites often serve as biological indicators of the health status of many fish species (Fiorenza et al., 2020). Recent research has revealed that cases of anisakiasis are rarely reported due to the absence of specific diagnostic methods and frequent misdiagnoses resulting from low levels of knowledge about the pathogenesis of the disease (Bao et al., 2019). The incidence of anisakiasis continues to increase. According to the quantitative risk assessment model, the prevalence of anisakiasis only in Spain is expected to increase from 500 to 7,700-8,320 cases per year (Bao et al., 2017).

The lack of data on sugar uptake mechanisms and their importance in the biology of this parasite highlights the need for further research.

## 2 Materials and methods

### 2.1 Analysis of genome organization and transcript variants

The genomic sequences for *fgt-1* (GenBank: MF069077], *fgt-2* (GenBank: MG557622), *fgt-3* [GenBank: MG557623], *fgt-5* [GenBank: MG210725], *fgt-9* (GenBank: MG557624) and *sweet-1* (GenBank: MG210740) were published previously (Łopieńska-Biernat et al., 2019a) and used in this study. The cDNA sequences of the three selected *A. simplex s. s.* facilitated glucose transporter genes were compared with genomic sequences available from the Wellcome Trust Sanger Institute BLAST server (http://www.sanger.ac.uk/cgi-bin/blast/submitblast/a_simplex) and GeneDB (http://www.genedb.org/Homepage/Asimplex) of the corresponding genes of other species: *Toxocara canis*, *Caenorhabditis elegans*, and *Homo sapiens*. Intron–exon junctions were manually detected (5′GT and 3′AG) using sequence alignment results obtained and images created using Lasergene MegAlign Pro software from DNASTAR, Inc.

### 2.2 3D structure modelling

The alignment of protein sequences of interest was performed using MUSCLE v.3.8.425 (Edgar, 2004). To approximate an accurate tertiary model of two selected *A. simplex* s. s. proteins, FGT1 (GenPept: AVV64024.1) and FGT3 (GenPept: AXS78307.1), several protein structure prediction servers were used: FOLDpro (Cheng and Baldi, 2006), I-TASSER (Zhang, 2008), 3DJigsaw (Bates et al., 2001), LOOPP (Vallat et al., 2009), Phyre2 (Kelley and Sternberg, 2009), and SwissModel (Arnold et al., 2006).

To assess the quality of the output models and select the top candidates, ResProx (Berjanskii et al., 2012), QMEAN (Benkert et al., 2009), and ModFOLD (McGuffin et al., 2013) were used.

The top three models for each protein were manually inspected to detect any unresolved secondary structures (e.g., α-helices) and to optimize the structures by removing steric clashes. The optimized top models were selected for presentation. Root means square deviation (RMSD) was used to measure the average atomic distances between two protein structures: one based on the *A. simplex* s. s. sequence and the second based on the sequence of *H. sapiens*, *T. canis*, and *C. elegans*, respectively.

Additionally, predicted tertiary structures of *A. simplex* s. s. sugar transporters were generated using AlphaFold 3 AI (Abramson et al., 2024) with default parameters (https://alphafoldserver.com). Input amino acid sequences were obtained from GenPept. Model confidence was evaluated using two AlphaFold-specific metrics: the per-residue predicted Local Distance Difference Test (pLDDT) and the predicted Template Modeling score (pTM).

### 2.3 Phylogenetic analysis of putative sugar transporters

**Figure 1:**
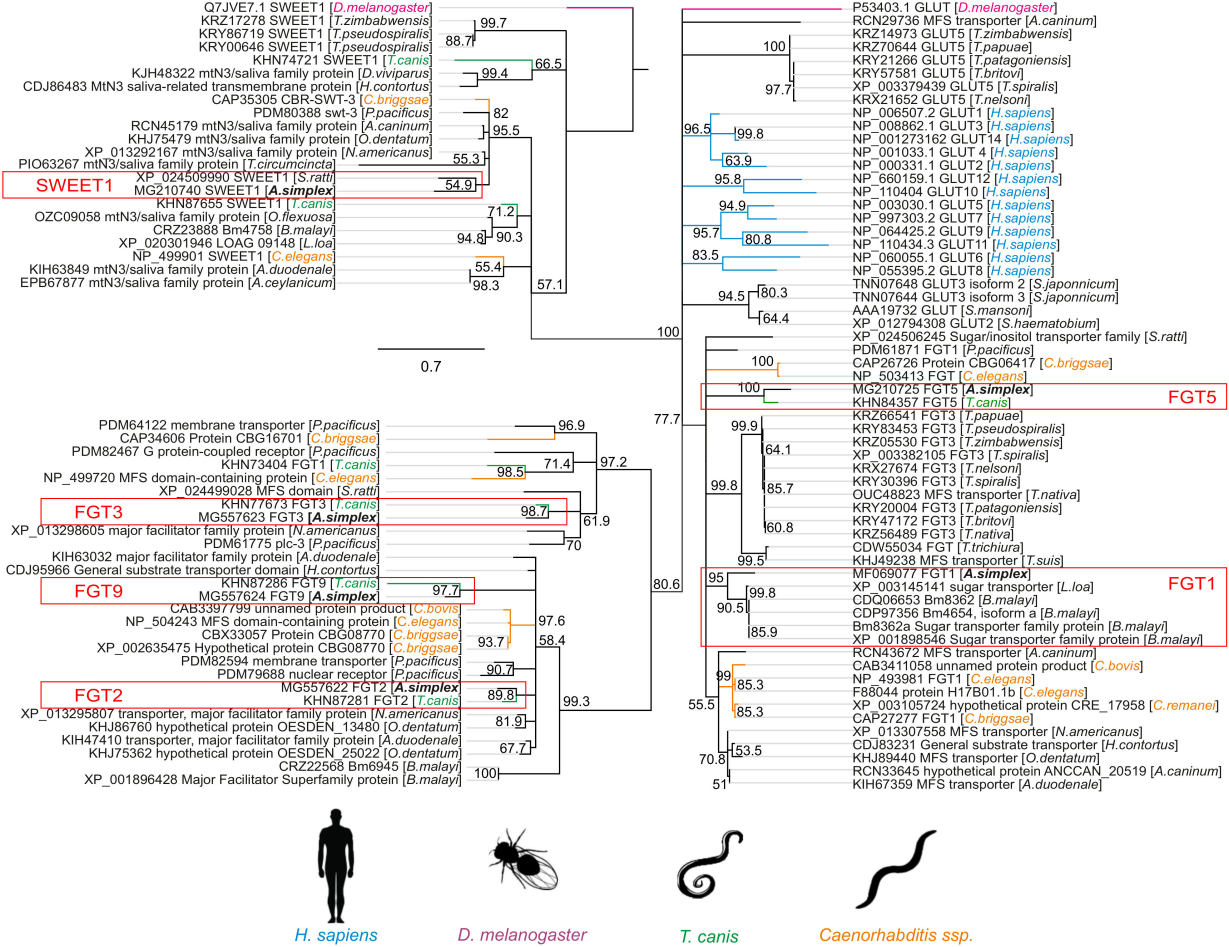
The phylogeny tree of the glucose transporter family. The figure shows a phylogenetic tree of facilitative glucose transporters (FGTs) and the “Sugars Will Eventually Be Exported Transporters” (SWEETs) of vertebrates, nematodes and insects. A rooted tree was constructed with the neighbor-joining method using MEGA ver. 7. The numbers on the internal branches are the bootstrap values (only >50% are shown). The sequences are labeled with data from GenBank, such as species name, name and accession number of the sugar transporters. The glucose transporters of *A. simplex* are indicated by the red frame. The sucrose transporters of *Drosophila melanogaster* are highlighted in purple, the GLUTs of *Homo sapiens* in blue, the transporters of *Toxocara canis* in green and those of *Caenorhabditis* spp. in orange.

A rooted tree was constructed using the neighbor-joining method in MEGA ver. 7 (Kumar et al., 2016) and tested with a bootstrap analysis of 500 replications. Sugar transporters from *A. simplex* s. s., *T. canis*, *H. sapiens*, *Caenorhabditis* spp., *Brugia malayi*, *Haemonchus contortus*, and the sucrose transporter from *Drosophila melanogaster* (introduced as an outgroup) were analyzed. These transporters belong to the solute carrier 2 (SLC2) family and SWEET1 family and were identified based on the GeneDB database (http://www.genedb.org/) with a BLASTP search cutoff E-value of 10^−^¹, using the GLUT1 amino acid sequence as the query. The species, sugar specificity, and accession numbers for each sequence are provided in **Figure 1**. The conserved domain sequences of predicted sugar transporter genes were confirmed using the HMMER program (http://hmmer.org). All sequences are available in the GeneDB database using the accession numbers indicated in the phylogenetic tree.

The scale bar represents 0.2 substitutions per amino acid site.

### 2.4 Parasites

The experiment was performed on L3 developmental stage *A. simplex* s. s. obtained directly from herring (*Clupea harengus membras*) and L4 developmental stage cultured *in vitro* from L3 (Iglesias et al., 2001). The fish were collected from the coastal waters of the southern Baltic Sea. L3 larvae were rinsed three times in sterile saline solution (0.9% NaCl) and washed for 30 min in bactericidal and fungicidal solutions. The larvae were then divided into two groups. The first group (L3) was directly used for *in vitro* culture with glucose. The second group was cultured to the L4 stage *in vitro* and then used to *in vitro* culture with glucose. Additionally, fifteen of the L3 larvae used in the experiment were subjected to taxonomic identification based on the amplification of the ITS1/ITS2 region of genomic DNA as described by (Polak et al., 2022). Genomic DNA required for identification was isolated using a commercially available kit according to the manufacturer’s instructions (Xpure™ Cell & Tissue, A & A Biotechnology, Gdynia, Poland).

#### 2.4.1 *In vitro* culture of L4 stage larvae

L3 larvae were cultured in RPMI-1640 medium (R8758, Sigma Aldrich, USA) acidified to pH 4 with 1M HCl, enriched with 20% fetal bovine serum (F7524, Sigma Aldrich), and 1% pepsin (P7125, Sigma Aldrich) in six-well culture plates (BD Biosciences, Poland) at 37°C with 5% CO_2_, following the method described by Iglesias et al. (2001). The *in vitro* culture to the L4 developmental stage lasted 6 days.

#### 2.4.2 *In vitro* culture of *A. simplex* s. s. with glucose

L3 and L4 larvae of *A. simplex* s. s. were cultured *in vitro* with glucose at concentrations of 0.1, 0.5, 2, 10, 15, and 20 mg per mL of culture medium (as described above). Ten parasites from each developmental stage were cultured in each of the mentioned glucose concentrations for 12 h and 24 h (240 larvae in total). In addition, L3 and L4 larvae of *A. simplex* s. s. without glucose were cultured as a control (40 larvae in total). Larvae (all alive) were taken from the cultures after 12 h and 24 h, and samples (24 treated and 4 controls; 10 larvae in each sample) were preserved at -80°C until the next stage of analysis. Four larvae from each sample were used for total RNA isolation, and the remaining larvae were used for biochemical analyses.

#### 2.4.3 *In vitro* culture of *A. simplex* s. s. L3 with ivermectin

The *in vitro* culture scheme was adopted from Stryiński et al. (2024). In brief, L3 larvae of *A. simplex* s. s. (4 individuals per concentration and time; 48 individuals in total); were cultured *in vitro* for 12 h with ivermectin [dissolved in 0.1% dimethylsulphoxide (DMSO); 45684, Sigma Aldrich, Poznan, Poland] at three concentrations: 6.25 (lowest), 12.5 (medium) and 25 (highest) μg/mL of culture medium. The control culture did not contain ivermectin (the medium had an addition of 0.1% DMSO). Larvae were examined under a stereo microscope after 3, 6 and 12 h (all the larvae died after that time) to test the efficacy of the anthelmintics. Larvae with no mobility or contractions of the ventriculus or with alteration of the cuticle were considered dead. After the culture, all L3 larvae were collected at a specific time point and drug dose (four larvae per samples) and subjected for RNA isolation and gene expression studies.

### 2.5 Total RNA isolation, cDNA synthesis and quantitative real-time PCR

Total RNA from all samples was isolated using the Total RNA Mini Plus kit (036-100, A & A Biotechnology, Gdynia, Poland) following the manufacturer’s instructions. cDNA was synthesized from 2 μg of RNA using oligo(dT) primers and reverse transcriptase from the TransScriba kit (4000-100, A & A Biotechnology, Gdynia, Poland). The resulting cDNA was stored at -20°C for further analysis.

The mRNA expression levels of the putative facilitated glucose transporter genes (*fgt-1*, *fgt-2*, *fgt-3*, *fgt-5*, *fgt-9*) and one Sugars Will Eventually be Exported Transporter (*sweet-1*) were determined in L3 and L4 stage larvae of *A. simplex* s. s. The quantitative real-time PCR was performed using a QuantStudio 3 System (Applied Biosystems, Foster City, CA, USA). The primers used in the experiment were designed using Primer3 v 0.4.0 (Untergasser et al., 2012) based on gene sequences from the GenBank database (http://www.ncbi.nlm.nih.gov/) (**Supplementary Table S1**), and the annealing temperatures were optimized for each primer. The real-time PCR reaction mixture contained 500 ng of cDNA, 5 μL of Sensitive RT HS-PCR Mix SYBR^®^ (2017-1000BM, A & A Biotechnology, Gdynia, Poland), 1000 nM of each primer, 0.2 μL of LoROX reference dye (A & A Biotechnology, Gdynia, Poland), and nuclease-free water to a final volume of 10 μL. Reactions were carried out in triplicate. Relative expression levels, presented as fold changes relative to the untreated control and normalized to the endogenous reference gene (*ef-1α*, with a relative quantification RQ = 1), were calculated using the comparative Pfaffl method (Pfaffl, 2001). The data are expressed as means ± standard deviation.

### 2.6 Biochemical analyses

#### 2.6.1 Preparation of larvae homogenates

L3 and L4 larvae from each sample were thawed, dried, and weighed. The larvae were then mechanically homogenized on ice with 0.65% NaCl (1/10 w/v). After homogenization, the larvae were centrifuged at 800 × g for 15 minutes at 4°C. The supernatants obtained were used to determine the content of glucose, trehalose, and glycogen, as well as to assess the activity of trehalase.

#### 2.6.2 Quantitative determination of glucose, trehalose, glycogen content, and trehalase activity

The glucose content was determined using the Glucose Oxidase Reagent Set (G7521, Pointe Scientific, Poland). During the procedure, glucose was oxidized by glucose oxidase to gluconate and hydrogen peroxide. Subsequently, phenol and 4-aminoantipyrine reacted with hydrogen peroxide in the presence of peroxidase to produce a quinoneimine dye, which was measured at 500 nm. The absorbance at this wavelength was proportional to the glucose concentration in the sample.

Trehalose content was determined by the enzymatic method described by Kienle et al. (1993), using trehalase from porcine kidney (1 U/mg of protein; T8778, Sigma Aldrich, Poland). Following quantitative hydrolysis of trehalose by trehalase, the released glucose was quantified using the Glucose Oxidase Reagent Set mentioned above.

Glycogen content was measured using the micro-method developed by Sølling and Esmann (1975). This method involves the precipitation of glycogen on Whatman filter paper, followed by degradation of the precipitated glycogen using amylo-glucosidase. The glucose released during this process was also determined spectrophotometrically using the Glucose Oxidase Reagent Set. The contents of glucose, trehalose, and glycogen were expressed in milligrams per milligram of tissue (mg/mg tissue).

Trehalase activity was assessed using a modified method by Dahlqvist (1984). One enzymatic unit was defined as the amount of enzyme that released 1 µmol of glucose at 37°C in one hour. At the end of the reaction, glucose content was quantified using the Glucose Oxidase Reagent Set. Trehalase activity was normalized to protein content and expressed as μmol/mg of protein. Protein concentration was determined using the Bradford method (Bradford, 1976).

All results presented are means ± standard deviation of three independent replicates.

### 2.7 Statistical analyses

A two-way analysis of variance (ANOVA) was performed using GraphPad Prism 8 software (GraphPad Software Inc., San Diego, CA, USA). The Shapiro–Wilk test was used to assess normality, and Levene’s test was applied to test for homogeneity of variance. All datasets met these assumptions and were analyzed using parametric methods. Differences between means were evaluated using Dunnett’s multiple comparisons test for gene expression data, and Tukey’s test for biochemical analysis data. Statistical significance was defined as a *p-*value ≤ 0.05.

## 3 Results

### 3.1 Characterization of *A. simplex* s. s. *fgt* and *sweet* genes and proteins

We obtained cDNA sequences of five putative facilitated glucose transporter genes (*fgt-1, fgt-2, fgt-3, fgt-5, fgt-9*) and one *Sugars Will Eventually be Exported Transporter* (*sweet-1*) from *Anisakis simplex* s. s.

**Figure 2:**
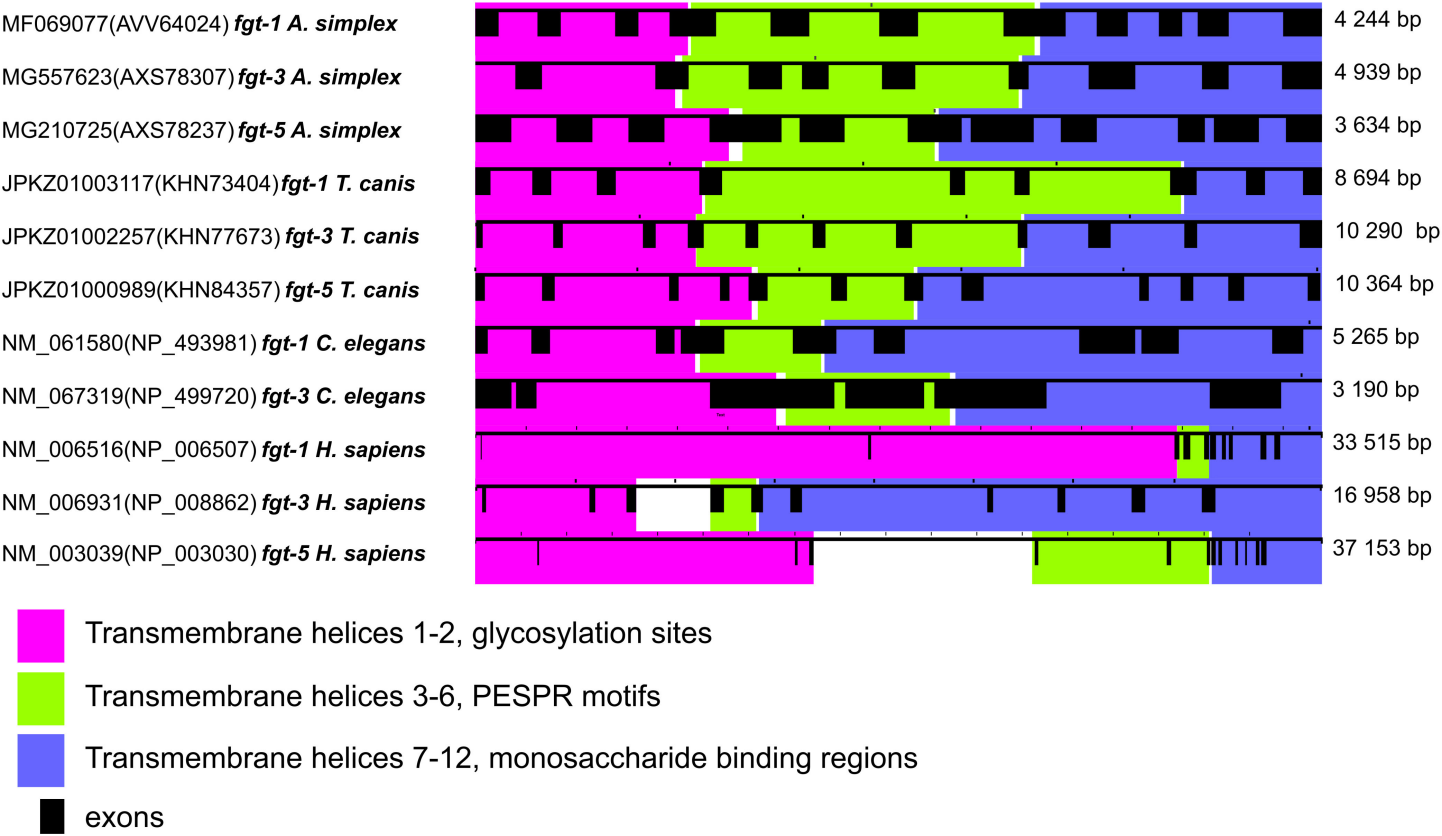
Genomic organization of selected facilitative glucose transporters. The genomic organization of the coding regions of *Anisakis simplex* glucose transporters is presented. The genomic organization of *Toxocara canis*, *Caenorhabditis elegans* and human glucose transporters are shown for comparison. Exons are represented by filled black boxes and introns by connecting lines. The size of the sequence is indicated in base pairs on the right-hand side of the figure. Exons are numbered from left to right and described in **Supplementary Material**. Transmembrane helices involved in the different functions of the proteins are highlighted in purple, green and blue.

Three *fgt* sequences identified in the *A. simplex* s. s. with significant homology to members of the mammalian GLUT family of glucose transporters were selected for further bioinformatical analyses. **Figure 2** shows the genomic organization of the coding regions (CDS, coding sequence) of *A. simplex* s. s. facilitated glucose transporters compared to their homologs from *T. canis*, *C. elegans*, and *H. sapiens*. Exons, introns, and transmembrane domains are annotated, indicating structural conservation. Detailed data about CDS and exons can be found in **Supplementary Table S2**.

**Figure 3:**
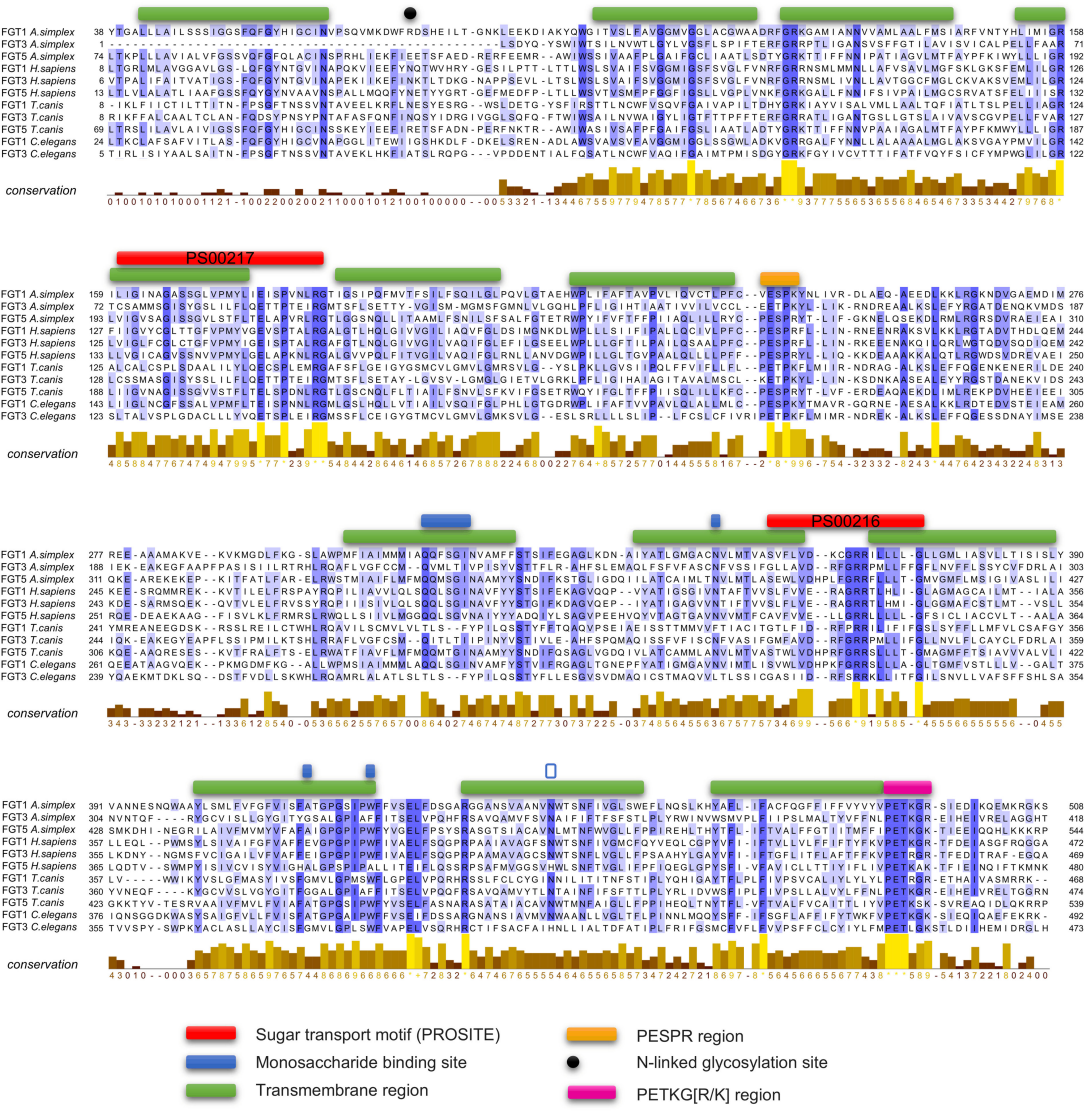
Alignment of members of the facilitative glucose transporters (FGT 1, 3, 5). The amino acid sequences of FGTs from *Anisakis simplex, Toxocara canis, Caenorhabditis elegans* (FGT 1,3*)* and humans were aligned with MAFFT. Relevant motifs between the glucose transporters of the selected organisms are highlighted and annotated in the figure legend. The degree of sequence conservation is also characterized. Sequence numbers are listed in **Figure 2**.

Multiple sequence alignment (MSA) revealed that FGT1, FGT3, and FGT5 transporters from *A. simplex* s. s. shared well-conserved residues with homologous sequences from *H. sapiens*, *T. canis*, and *C. elegans* (**Figure 3**). Important functional residues, particularly in transmembrane region, such as G36, R71, E81 and A167 (for FGT3), were highly conserved, supporting the classification of these proteins as glucose transporters. In addition, both characteristic Major Facilitator Superfamily (MFS) signature motifs, PS00216 and PS00217, which are essential for facilitated glucose transport, were detected in the sequences of *A. simplex* FGT proteins, further supporting their functional classification as glucose transporters. Structural analysis also revealed the presence of conserved functional motifs, namely PESPR and PETKG[R/K], within the predicted tertiary structures of *A. simplex* s. s. glucose transporters. These motifs are crucial for the translocation of monosaccharides across the membrane (**Figure 3**).

**Figure 4:**
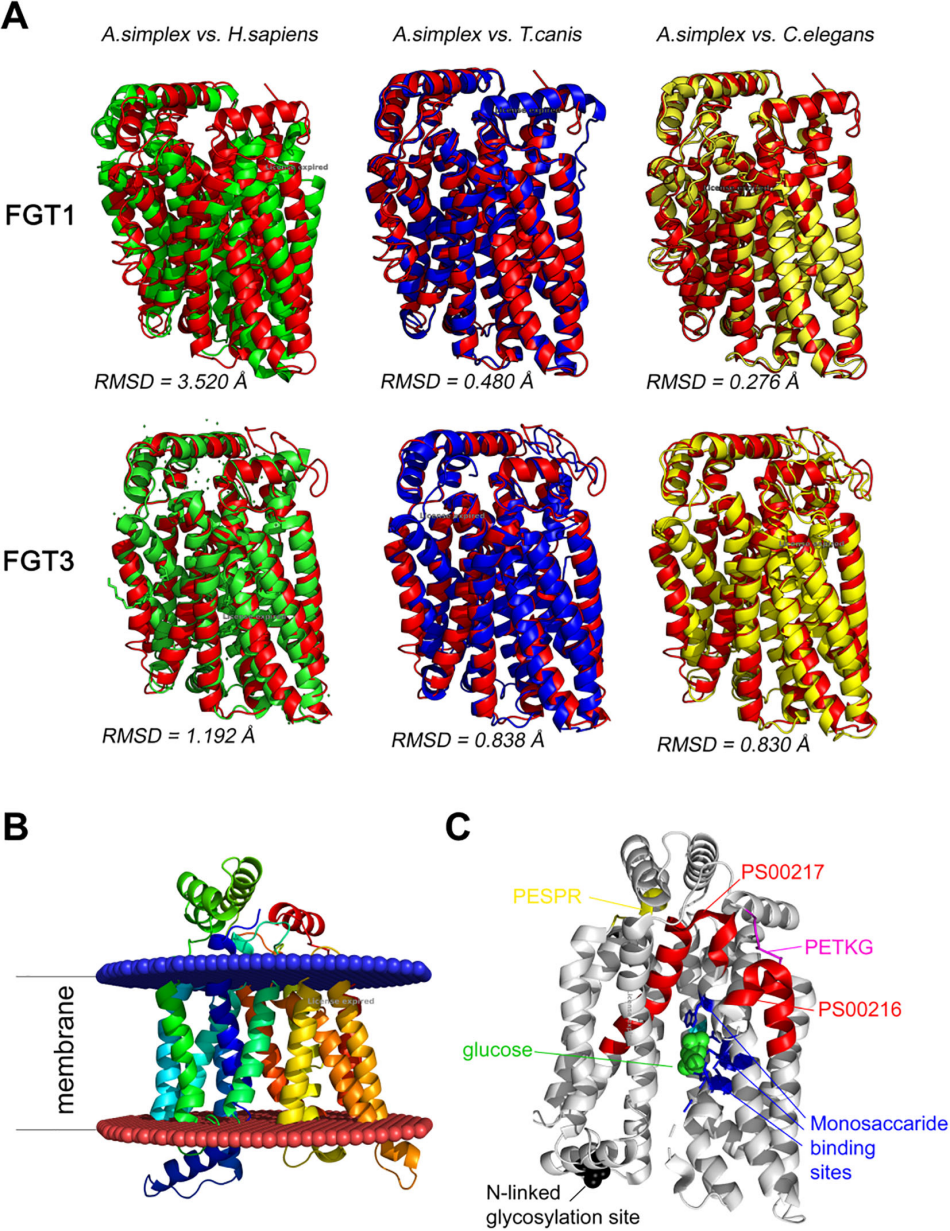
**(A)** Predicted tertiary structures of FGT1 and FGT3 of *A. simplex*. The structures are color-coded (structural alignment) for the respective species: *A. simplex* s. s.—red, *H. sapiens*—green, *T. canis*—blue, *C. elegans*—yellow. The root mean square deviation (RMSD) value indicates the average deviation between the corresponding atoms of two proteins: smaller value of RMSD means that compared structures are more similar. **(B)** Visualization of twelve transmembrane regions in FGT1 of *A. simplex*. The region above the blue lipid layer is the extracellular region, and the region below the red lipid layer is the intracellular region. **(C)** Visualization of the tertiary structure of FGT1 in *A. simplex* with indication of characteristic motifs (PESPR, PETKG, PS00216, PS00217), N-linked glycosylation site and monosaccharide binding site.

The predicted tertiary structures of *A. simplex s. s.* FGT1 and FGT3 and their structural similarity to homologous proteins from other species are shown in **Figure 4**. The root mean square deviation (RMSD) value indicates the average deviation between the corresponding atoms of two proteins: the smaller the RMSD value, the greater the structural similarity. For FGT1, the RMSD values indicated the highest structural similarity to the glucose transporters of *C. elegans* and *T. canis*, respectively (RMSD 0.276 Å and 0.480 Å), followed by *H. sapiens* (RMSD 3.520 Å). In contrast, FGT3 showed slightly higher RMSD values for *C. elegans* and *T. canis* predicted structures (0.830 Å and 0.838 Å, respectively), and lower for *H. sapiens* (1.192 Å) reflecting minor structural differences between nematode and human glucose FGT3 transporters (**Figure 4A**).

Visualizations of the FGT1 model revealed the presence of twelve transmembrane domains (TM1-TM12), N-linked glycosylation sites, monosaccharide binding sites, and characteristic sugar transporter motifs, including PESPR, PETKG, PS00216, and PS00217 (**Figures 4B, C**). Visualizations of the predicted tertiary structures of selected *A. simplex* s. s. sugar transporters, generated using AlphaFold 3 AI, along with confidence estimates based on pLDDT and pTM scores, are provided in **Supplementary Figure S1**.

Phylogenetic analysis grouped *A. simplex* s. s. glucose transporters into three distinct clusters (**Figure 1**). Bootstrap values (only >50% are shown) were used to assess the robustness of the phylogenetic groupings. Values greater than 70% were considered indicative of reliable clades. It should be noted that bootstrap support reflects the confidence in the branching order rather than the actual sequence similarity or evolutionary distance between taxa.

Two clusters were associated with glucose transporters specific to parasitic Clade III nematodes, particularly *T. canis* (with paralogs showing 90–100% similarity), *Loa*, or *B. malayi*. Moreover, FGT2, FGT3, and FGT9, with a bootstrap value of 80,6%, reflects the higher similarity of 3D structure of FGT3 to *H. sapiens* GLUTs, rather than FGT1 (77,7%). SWEET1 clustered with *Strongyloides ratti*, a clade IV nematode.

### 3.2 Expression of *fgt* and *sweet* in *A. simplex* s. s.

**Figure 5:**
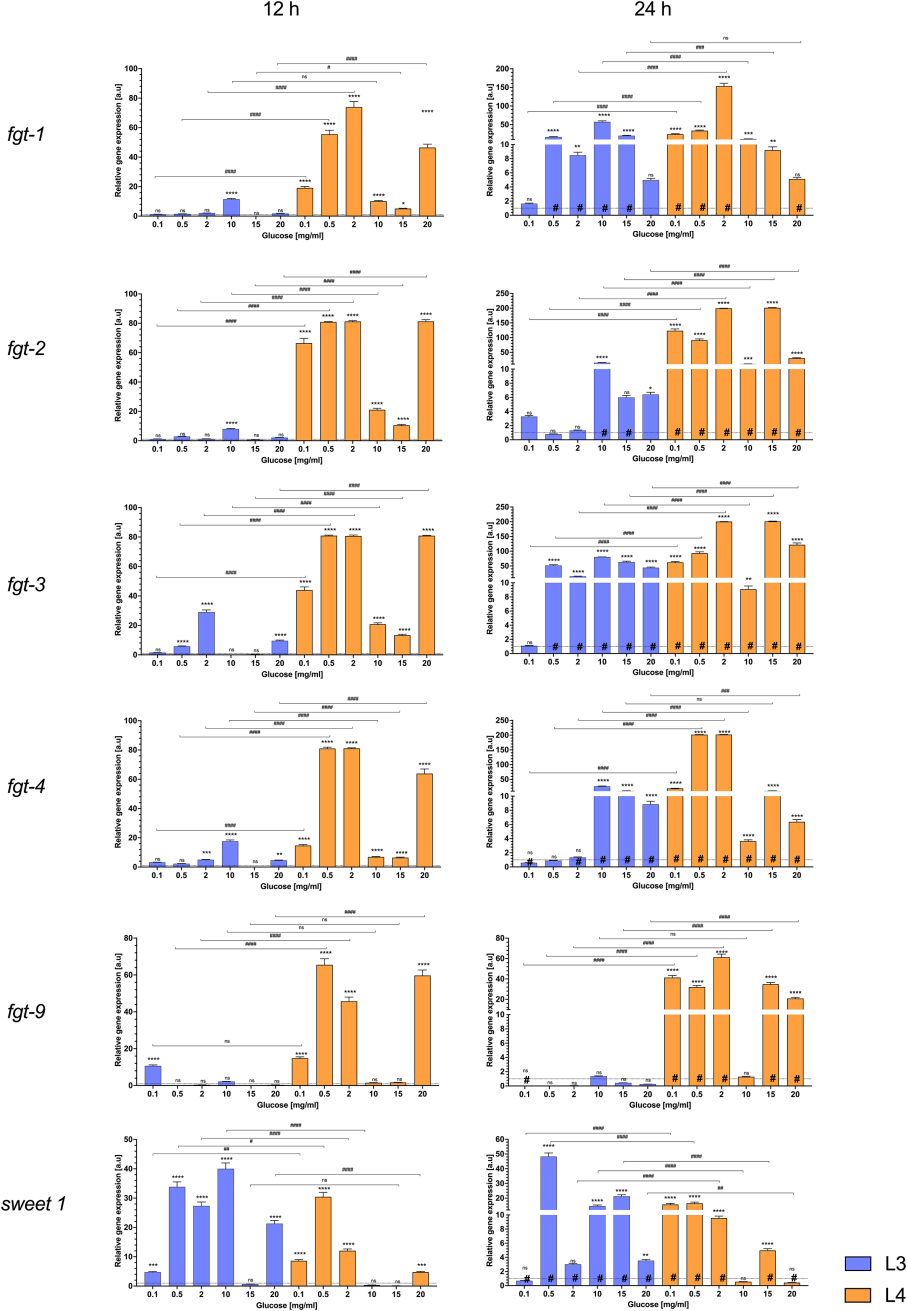
The mRNA expression of *fgt 1, 2, 3, 5, 9*, and *sweet 1* genes in L3 larvae (blue bars) and L4 larvae of *A. simplex* (orange bars) exposed to different glucose concentrations (0.1, 0.5, 2, 10, 15, 20 mg/mL culture medium) during 12 h (left panel) and 24 h (right panel) of an *in vitro* culture. Depicted values indicate the means of three replicates ± SD. The data were presented as the fold change in gene expression normalized to a reference gene *ef1-α* and relative to the untreated control (relative quantification RQ = 1). A two-way ANOVA analysis was performed and the differences between means (glucose concentrations [*], time of the culture [#] and larvae developmental stages [^###^]) were assessed by Dunnett’s multiple comparisons test. p Values were considered statistically significant, where 0.0332 (*, ^#^), 0.0021 (**, ^##^), 0.0002 (***, ^###^), and <0.0001 (****, ^####^). # - significance with p value ≦̸ 0.05; ns, non-significant results.

Six glucose transporter genes (*fgt-1*, *fgt-2*, *fgt-3*, *fgt-5*, *fgt-9*, and *sweet-1*) showed distinct expression responses to external glucose in L3 and L4 larvae. Overall, glucose availability had a pronounced impact on transcript levels, with significant differences (Dunnett’s test, p ≤ 0.05) observed at various concentrations and two time points (**Figure 5**).

In infective L3 larvae (**Figure 5**, blue bars), many transporter genes were upregulated by the presence of glucose, but the extent of the change often depended on glucose concentration and incubation time. After 12 hours, there were few significant differences compared to controls. For example, the relative gene expression of *fgt-3* and *fgt-5* increased significantly above the glucose-free control at glucose concentrations of 0.5, 2, 20 mg/mL and 2, 10, 20 mg/mL, respectively (**Figure 5**). In addition, the expression of some genes stagnated at the highest dose (20 mg/mL) or even slightly decreased to baseline. This is evident in cases such as *fgt-9*, which showed its strongest induction at the lowest glucose levels (significantly higher than the control at 0.1 mg/mL), but at 20 mg/mL its expression was no longer different from the control (likely reflects an adaptive regulation mechanism, rather than classic feedback attenuation, in the presence of excess glucose).

After 24 hours, the expression differences in L3 became clearer. Several transporters that were only slightly induced after 12 hours showed increased expression after 24 hours. *Fgt-1*, for example, did not respond strongly at baseline (significant upregulation at one concentration after 12 hours), but after 24 hours of incubation its expression was so upregulated that it was significantly different from the control at some mid-range concentrations (e.g. 0.5–15 mg/mL), as well as after 12 hours of culture. This suggests a stage-specific, time-dependent regulation, with initial glucose sensing in L3 by *sweet-1*, followed by upregulation of *fgt* genes upon prolonged exposure. The expression of *fgt* generally increased with glucose concentration and duration of culture (24h). In the L3 larvae, only *fgt-9* showed slight changes in expression after 24 hours. In addition, it can be seen that its expression was completely silenced after 24 hours compared to the control and the 12-hour culture (**Figure 5**).

In the more advanced L4 larvae, the expression of the transporter genes responded more evenly and robustly to the different glucose treatments. Almost all glucose concentrations tested (0.1–20 mg/mL) resulted in significant changes in expression of the glucose transporters tested up to 12 hours. For example, *fgt-1, fgt-2, fgt-3, fgt-5, fgt-9* and *sweet-1* were upregulated in the presence of glucose, often showing a significant increase even at the lowest concentration (0.1 mg/mL) compared to starved controls (*p*-value ≤ 0.05). A decrease in expression was observed at concentrations of 10 and 15 mg/mL after 12 hours of culture in L4 larvae, while the differences in expression of *fgt-9* and *sweet-1* were not statistically significant at these glucose concentrations compared to controls. In contrast to L3, L4 larvae tended to show earlier saturation of the response: additional glucose did not always lead to significantly higher expression once a threshold was reached or even decreased in the case of the previously mentioned concentrations of 10 and 15 mg/mL. In practice, this meant that the L4 transporters were either “off or on”; a small amount of glucose triggered maximal (or near maximal) expression and further increases in glucose concentration up to 20 mg/mL resulted in only modest or no additional effects (*fgt-2, fgt-3, fgt-9*). It is noteworthy that the increased expression in L4 was often maintained over 24 hours. In contrast to L3, there was stable, high expression over time. Genes such as *fgt-1, fgt-2, fgt-3* and *fgt-5* remained highly expressed (approximately twofold change in a.u.) at all glucose levels after 24 hours (although expression was still well above control at each concentration tested after 24 hours). This sustained upregulation suggests that L4 larvae keep their glucose uptake machinery active as long as glucose is available, consistent with their role as actively feeding parasites. *Sweet-1*, a putative sugar transporter from a different family, responded significantly at both stages. However, *sweet-1* transcripts in L3 increased progressively with glucose dose after 12 hours of culture (significantly at each step), while this expression gradually decreased after 24 hours. In addition, *sweet-1* transcripts in L4 appeared to reach a high expression level early on (significant increase at 0.1, 0.5 and 2 mg/mL with minimal further increase or even decline) (**Figure 5**).

In summary, L4 larvae generally exhibit higher and more sustained transporter gene expression in response to glucose than L3 larvae. L3 transporters were inducible but showed transient or concentration-dependent, overall low expression (with expression of some genes peaking at intermediate glucose levels and then increasing at high glucose concentrations for up to 24 hours), which may indicate stronger feedback control at the infective stage. In contrast, L4 responded to even low glucose concentrations with a strong upregulation of transporters and largely maintained these levels (24 h), indicating a stage primed for continuous nutrient uptake. All described expression changes were statistically significant (*p*-value ≤ 0.05) compared to controls, emphasizing that glucose availability is an important regulator of transporter gene expression in both stages.

### 3.3 Glucose, trehalose, and glycogen content, and trehalase activity in *A. simplex* s. s.

The glucose metabolism of *A. simplex s. s.* larvae was investigated by measuring the internal content of free glucose, trehalose, glycogen and the activity of trehalase (the enzyme that hydrolyses trehalose) after 24 hours in different glucose concentrations. Clear differences were observed between L3 and L4 larvae in the way they accumulate and utilize sugars under different glucose concentrations in the environment.

**Figure 6:**
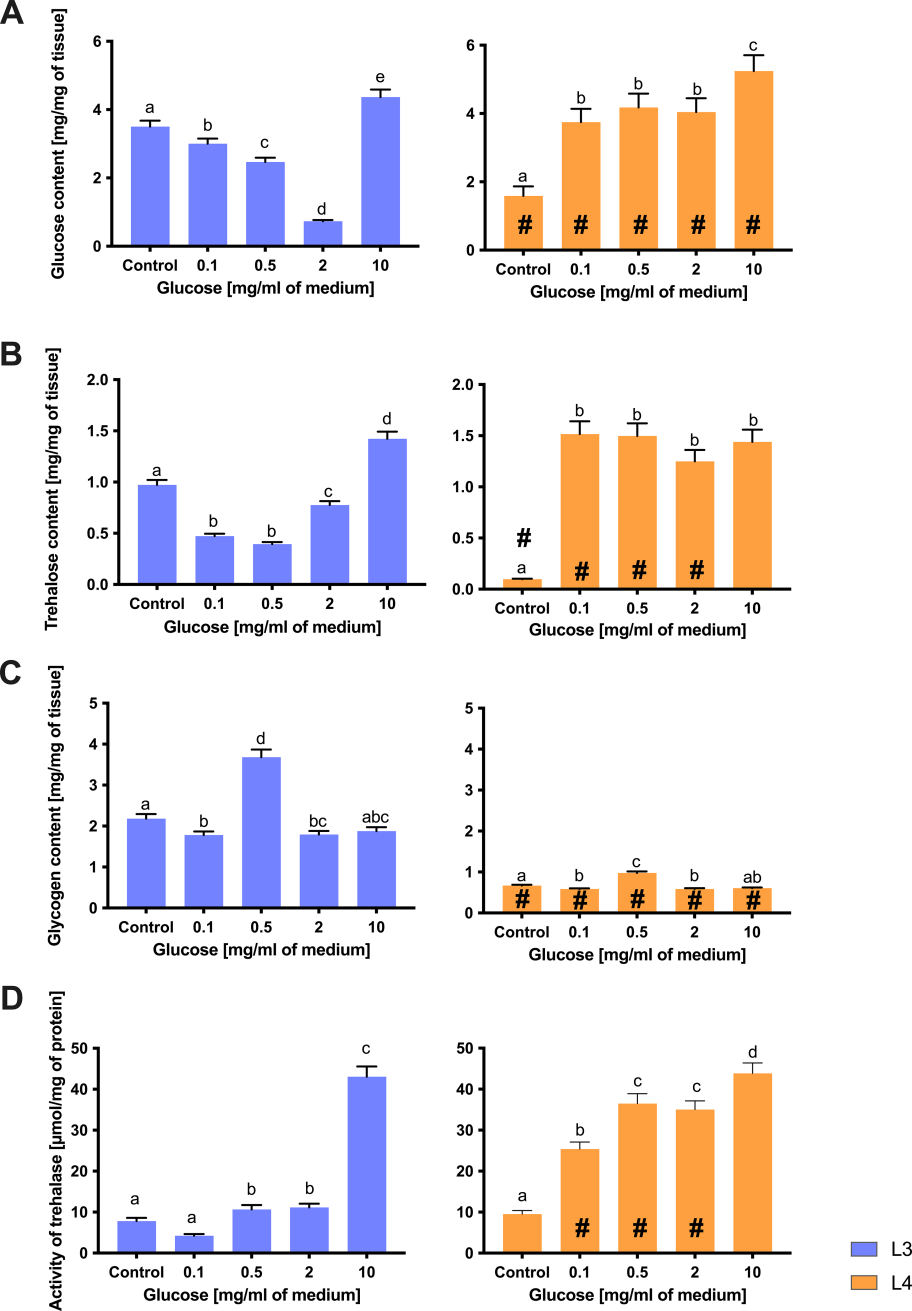
The content of glucose **(A)**, trehalose **(B)**, and glycogen **(C)**, as well as the activity of trehalase **(D)** in L3 larvae (blue bars) and L4 larvae of *A. simplex* (orange bars) exposed to different glucose concentrations (0.1, 0.5, 2, 10 mg/ml culture medium) during 24 h of an *in vitro* culture. Depicted values indicate the means of three replicates ± SD. A two-way ANOVA analysis was performed and the differences between means (glucose concentrations [^a,b,c^], and larvae developmental stages [#]) were assessed by Dunnett’s multiple comparisons test. Bars with different letters indicate statistically insignificant differences (p value ≦̸ 0.05). # - significance with p value ≦̸ 0.05.

The amount of free glucose in larval tissues decreased with the availability of external glucose in L3 and increased in L4 (**Figure 6A**). In L3 larvae, the glucose content decreased gradually and proportionally to the mean glucose concentration. A decrease in internal glucose content was observed in larvae cultured at glucose concentrations ranging from 0.1 to 2 mg/mL (all significantly lower than the control without glucose), with further significant increases in internal glucose content at 10 mg/mL glucose in the medium. At 2 mg/mL, L3 larvae had reached the lowest glucose levels in their tissues (approximately a 4-fold decrease compared to control), with each concentration yielding a value that was statistically different from the others (indicating a dose-dependent accumulation). L4 larvae, on the other hand, showed rapid saturation of glucose uptake. Even a minimal glucose intake (0.1 mg/ml) led to a significant increase in tissue glucose content in L4 (2-fold compared to control), but higher concentrations did not lead to a corresponding increase. Instead, tissue glucose of L4 stagnated at low doses: from 0.1 to 2 mg/mL there was little further change (no significant differences between these treatments), and only at the very highest concentration (10 mg/mL) was a further modest increase observed. Thus, L3 accumulated free glucose in a more linear, dose-dependent manner, whereas L4 reached near-maximal internal glucose levels at low external inputs (suggesting that L4 tissue rapidly equilibrates or regulates free glucose to a limit).

Trehalose, an important storage sugar, was abundant in both stages but responded differently to glucose feeding (**Figure 6B**). L3 larvae accumulated trehalose in a dose-dependent manner, reflecting their pattern of glucose uptake. The trehalose content in L3 decreased significantly with increasing glucose concentration in the medium. At 0.1–0.5 mg/mL, the trehalose content decreased to a new stable level (both concentrations yielded significantly less trehalose than the control, although they did not differ from each other), indicating an initial consumption of trehalose reserves. With further increases in glucose intake to 2 mg/mL and especially 10 mg/mL, trehalose concentrations increased further (each step showed an additional significant increase). At the highest glucose concentration, trehalose content was higher in L3 than in controls, which could indicate an active conversion of external glucose into trehalose stores (**Figure 6B**). L4 larvae, on the other hand, showed an initial increase in trehalose that quickly levelled off. Even a small amount of glucose (0.1 mg/mL) supplied to the L4 cultures resulted in a large increase in trehalose (well above control), but increasing the glucose concentration beyond this did not result in a further significant increase in trehalose concentration. All glucose-treated L4 groups accumulated a similar amount of trehalose by 24 hours (with no statistical differences between the 0.1, 0.5, 2 or 10 mg/mL conditions), suggesting that the L4 larvae rapidly synthesize trehalose to a maximum or optimal level and then maintain it (**Figure 6B**).

Glycogen, another important energy reserve, showed a complex response, especially in L3 (**Figure 6C**). In L3 larvae, glycogen content initially increased at low glucose exposure (0.5 mg/mL), but then decreased at the highest concentrations (2, 10 mg/mL). After 24 hours without glucose, L3 had a moderate baseline glycogen level. The addition of 0.1 mg/mL glucose resulted in a significant decrease in glycogen stores (compared to control), and a further jump was observed at 0.5 mg/mL. This moderate glucose level resulted in the highest glycogen content measured in L3. However, when external glucose increased above 2 mg/mL, no further glycogen accumulation occurred. At glucose concentrations of 0.1 and 2 mg/mL, the glycogen content of L3 was well below the peak value of 0.5 mg/mL (although still below the control value), and at a glucose supplementation of 10 mg/mL, the glycogen content had fallen back to near control values (and was no longer significantly different from the group without glucose). This resulted in a bell-shaped trend: L3 glycogen peaked at intermediate glucose availability and then dropped at excess glucose concentrations. A likely explanation is that extremely high external glucose shifts L3 metabolism to other pathways (e.g., redirecting glucose to trehalose synthesis) rather than glycogen (which would be possible if trehalose concentration were considered; **Figure 6B**). In L4 larvae, glycogen content was comparatively stable and less responsive to glucose supply. We observed a modest increase in L4 glycogen at intermediate glucose doses (0.5 mg/mL), as reflected by a significant difference between the control and treated groups. However, unlike L3, glycogen levels in L4 did not fluctuate greatly across the range of glucose concentrations – there were no sharp increases or decreases. At 10 mg/ml, the glycogen content in L4 showed no change compared to the control. In fact, the glycogen content of L4 remained within a relatively narrow range for all glucose treatments, and the increase at 0.5 mg/mL of medium glucose was small. This indicates that the L4 larvae tightly regulate their glycogen stores, probably because they start near a storage maximum. Excess external glucose was not efficiently converted to additional glycogen in L4, presumably because these larvae had less “room” for additional glycogen or prioritized maintaining the existing balance between trehalose and glycogen (**Figures 6B, C**).

The activity of trehalase, the enzyme that converts trehalose to glucose, was directly related to the glucose level in the environment (**Figure 6D**). Under glucose deprivation, trehalase activity was relatively high in both L3 and L4 larvae, indicating an active turnover of trehalose to provide glucose as an energy source when no external source was available. After glucose administration, trehalase activity increased significantly in both stages (compared to control). This increase was more pronounced at higher glucose concentrations. L3 larvae showed a generally low trehalase activity at the beginning and a strong increase in trehalase activity with glucose supplementation of 10 mg/mL, whereby the activity was fourfold higher than that of the control. In L4 larvae, trehalase activity generally increased when external glucose was supplied. Contrary to typical physiological expectations, trehalase activity increased at higher glucose concentrations. This unexpected pattern suggests a possible role for trehalase in balancing intracellular sugar pools or regulating trehalose turnover even under glucose-rich conditions, possibly as part of a broader metabolic adaptation strategy in *A. simplex* larvae. Of note is the stage-specific difference that L3 exhibited a wider dynamic range in trehalase activity (from low in control to very high in high glucose), whereas the trehalase activity of L4 increased rapidly even at the lowest concentration of added glucose (**Figure 6D**).

This again suggests a novel tendency for L4 to maintain high trehalase activity in the presence of high internal glucose concentrations, probably because L4 already has substantial trehalose reserves that it constantly utilizes and replenishes, whereas L3 either uses trehalose aggressively when hungry or stops using it when external food is supplied.

## 4 Discussion

Sugar transporters in helminths differ significantly from those in their mammalian hosts. *Schistosoma mansoni*, a parasitic trematode, possesses four glucose transporters, with two facilitating glucose diffusion. These belong to two distinct evolutionary clusters: one related to vertebrate and insect class I transporters, and another specific to parasitic Platyhelminthes, which are considered evolutionarily younger and exhibit slower rates of molecular evolution (Cabezas-Cruz et al., 2015). In *S. mansoni*, SGTP1 and SGTP4, located on opposite sides of the tegument, are essential for glucose uptake and parasite survival in the mammalian host (Krautz-Peterson et al., 2010). These differences between helminth and host sugar transporters may present potential targets for anti-parasitic drug development (Landfear, 2010).

Sugar uptake and efflux are fundamental processes for cell growth and metabolism in multicellular organisms. The FGT (facilitated glucose transporter) and SWEET (Sugars Will Eventually be Exported Transporters) families act as uniporters, facilitating sugar movement across membranes (Chen et al., 2015). In trematodes like *S. mansoni* and cestodes like *Echinococcus granulosus*, glucose can cross the tegument, supported by the presence of transporter proteins (Cabezas-Cruz et al., 2015; Kashiide et al., 2018). *In vitro* studies have shown that glucose-rich media enhance growth in *Hymenolepis diminuta*, highlighting glucose’s importance for cestode development (Graham and Berntzen, 1970; Kashiide et al., 2018).

In *C. elegans*, the FGT1 transporter exhibits broad substrate specificity, transporting not only glucose but also fructose, galactose, and mannose (Kitaoka et al., 2013). Silencing of *fgt-1* and *sweet-1* has been shown to extend lifespan by limiting glucose uptake and suppressing glycolysis (Feng et al., 2013), opening potential avenues for parasite control strategies. The identification of FGT isoforms and SWEET transporters in *A. simplex s. s.* provides new insights into sugar uptake mechanisms in parasitic nematodes and raises the question of whether trehalose, a key disaccharide, may also originate from exogenous sources. To date, protein transporters in *A. simplex* had not been functionally described, and our findings open new research directions.

In filarial nematodes, nutrient uptake occurs primarily through the cuticle, as most species possess a non-functional intestine. Numerous studies have demonstrated that the cuticle/hypodermis complex supports saturable, stereoselective, and competitively inhibitable nutrient transport, indicating the presence of specific carrier systems (Komuniecki and Harris, 1995; Thompson and Geary, 1995). The hypodermis in these species contains enzymes linked to amino acid transport, while the intestine lacks such metabolic machinery. In contrast, the surface membrane of filariae lacks the dynamic absorption properties observed in parasitic cestodes and trematodes (Howells, 2007).

Although transcuticular absorption in intestinal nematodes is less well-established, some evidence supports its existence. Early studies on *Ascaris suum* indicated minimal glucose absorption *in vitro* (Castro and Fairbairn, 1969), but later research demonstrated low-level uptake via the cuticle (Fleming and Fetterer, 1984). More convincingly, other intestinal nematodes such as *Haemonchus contortus* and *Parascaris univalens* can actively absorb glucose from the environment, even when pharyngeal ingestion is inhibited by ivermectin treatment (Geary et al., 1993; Dube et al., 2022). This supports the idea that the cuticle may serve as a significant route for glucose uptake under certain physiological conditions.

Consistent with these findings, our study revealed that expression of glucose transporter genes (*fgt-1, fgt-2, fgt-3, fgt-5*) was maintained or even upregulated, by up to 100-fold, in *A. simplex* L3 larvae treated with ivermectin, despite their non-functional intestine (**Supplementary Figure S2**). This observation strongly supports the hypothesis that transcuticular glucose absorption occurs in this species and may be critical for parasite survival during non-feeding or developmentally arrested stages.

Most molecular characterizations of helminth transporters focus on trematodes, where GLUT1 plays a key role in glucose metabolism (Macheda et al., 2005). In *E. multilocularis* and *S. mansoni*, GLUT1 shows high glucose transport activity (Cabezas-Cruz et al., 2015; Kashiide et al., 2018; Amahong et al., 2021). Helminth glucose transport is primarily mediated by GLUT (SLC2A) and SGLT families. GLUTs, which are Major Facilitator Superfamily (MFS)-type passive transporters, facilitate the diffusion of glucose and other hexoses across cell membranes. While bioinformatic analyses have identified many potential sugar transporters in *A. simplex* (Łopieńska-Biernat et al., 2019a; Stryiński et al., 2019, 2022) (**Supplementary Figure S3**), our study shows that only *fgt-1* and *fgt-3* were functionally expressed in both larval stages (**Figure 5**, 24h). This is in line with findings in *C. elegans*, where only FGT-1A shows clear glucose transport activity (Kitaoka et al., 2013).

Alignment of *C. elegans* FGT sequences with human GLUT1 highlights the presence of charged residues in the seventh transmembrane domain (TM7) of nematode proteins, contrasting with mammalian GLUTs, where such residues are absent. This suggests that the lack of central charged residues may be a hallmark of functional passive glucose transporters. All GLUT family members share common features: 12 transmembrane domains and conserved sugar transporter motifs (Sun et al., 2012). An A-motif, involved in salt bridge formation, was also identified in *A. simplex* between the fourth and fifth transmembrane domains (TM4–TM5) and the eighth and ninth transmembrane domains (TM8–TM9), as in human transporters (Drew et al., 2021). The A-motif is a conserved amino acid sequence characteristic of MFS transporters. It typically occurs in cytoplasmic loops and contributes to conformational transitions by forming reversible salt bridges. These interactions are crucial for substrate binding and release during the transport cycle, and their presence supports a conserved transport mechanism across species.

In the era of AI–driven advances in structural biology, this study also employed one of the latest tools, AlphaFold 3, to predict the three-dimensional structures of sugar transporter proteins from *A. simplex* s. s. The resulting models revealed well-folded transporter architectures with clearly defined transmembrane domains (**Supplementary Figure S1**). High pIDDT values, particularly within transmembrane helices, indicate strong local confidence in the predictions and support their potential functional relevance. Moreover, pTM scores exceeding 0.5 across almost all analyzed proteins (except FGT 2) suggest that the overall predicted folds are likely to approximate native conformations, providing a robust foundation for future comparative and functional studies.

In our structural analysis of *A. simplex* s. s. glucose transporters, several additional conserved features were annotated, including the PESPR region, which may serve as a functional analog to the A-motif, and is also implicated in conformational switching. Moreover, we identified canonical sugar transporter signatures PS00216 and PS00217, N-linked glycosylation sites, and conserved monosaccharide binding residues (**Figure 4B**). Together, these motifs highlight the evolutionary conservation and structural integrity of the *A. simplex* s. s. GLUT-like proteins.

Despite evidence of broad substrate usage in mammals, the physiological specificity of many glucose transporter isoforms remains unclear. For example, GLUT5 is specific for fructose but is expressed in tissues with low fructose exposure, such as the brain (Drew et al., 2021). Transporters must balance substrate specificity with weak binding to enable high turnover. The A-motif and its associated salt bridges likely contribute to the energy barrier modulation during transport, both in passive and active MFS transporters (Drew et al., 2021).

In *C. elegans*, FGT-1 is the only known glucose transporter and is localized to the basolateral membrane of intestinal cells, mediating diffusion-based glucose uptake (Thorens and Mueckler, 2010; Kitaoka et al., 2013). SGLT-type transporters responsible for glucose uptake from the intestinal lumen (i.e., luminal glucose uptake) in mammals have not yet been identified in nematodes (Suzuki et al., 2022). In *A. simplex* s. s., our results suggest that this function may be taken over by specific FGT isoforms, particularly FGT1 and FGT3, which were functionally expressed in larval stages and are likely responsible for mediating glucose acquisition either through the intestinal epithelium in L4 or transcuticularly in L3. However, this proposed localization, and functional differentiation requires confirmation through future immunolocalization studies.

Sequence similarity network analyses classify human GLUTs into three classes, with class I including GLUT1–5, 7, 9, 11, and 14 (Jia et al., 2019). All five FGT isoforms identified in *A. simplex* s. s. cluster within class I. These proteins are glycosylated, with N-glycosylation sites located in the exofacial loop between TM1 and TM2. The observed expression dynamics vary by developmental stage. Environmental conditions and host dependence may influence transporter gene regulation in *A. simplex*, distinguishing it from *C. elegans*. Further research is needed to determine whether the suppressed transporter expression in L3 confers advantages in lifespan or stress adaptation, as seen in *C. elegans*.

It remains challenging to explain why certain *fgt* isoforms exhibit markedly increased or decreased expression during particular developmental stages in *A. simplex* s. s. Comparative genomic and functional studies across parasitic nematodes will be essential to understand how transporter diversity contributes to physiological specialization.

Understanding the molecular basis for sugar transport is thus a fundamental question in biology, with important medical applications in parasitic nematodes (Mitrovic et al., 2023). Differences in glucose transport efficiency and specificity among parasitic species could form the basis for the rational design of Anisakis-specific glucose transporter inhibitors, offering a promising strategy for targeted anti-parasitic drug development.

## 5 Conclusions

The identification and expression analysis of facilitated glucose transporters and SWEET-family transporters in *A. simplex* s. s. provides valuable insights into sugar metabolism in parasitic nematodes. These findings deepen our understanding of glucose transport regulation in distinct larval stages and suggest that transporter expression is tightly linked to the nutritional environment and developmental status.

Differential expression patterns between L3 and L4 stages highlight distinct metabolic strategies: L3 larvae display more dynamic, concentration-dependent transcriptional responses, while L4 larvae exhibit rapid, stable activation of glucose transporter genes. These physiological differences likely reflect adaptations to differing host environments and nutritional access. Specifically, the observed divergence in transporter usage between larval stages may stem from a developmental transition in nutrient uptake strategy, from predominantly cuticular glucose absorption in L3, which lacks a fully functional intestine, to intestinal glucose transport in L4, where the intestine becomes active and physiologically competent. This shift in nutrient acquisition pathways is further supported by our findings of functional transmembrane glucose uptake in larvae with inactive intestines, likely mediated via transcuticular transport. Such a mechanism may be critical for parasite survival during non-feeding stages and represents a potential vulnerability for therapeutic intervention.

In summary, glucose availability strongly influences *fgt* and *sweet* genes expression and carbohydrate storage in *A. simplex* s. s. larvae. Given the divergence of *A. simplex* s. s. glucose transporters from mammalian counterparts, these proteins may serve as viable targets for the rational design of anthelmintic drugs aimed at disrupting parasite metabolism and survival.

## Data Availability

The original contributions presented in the study are included in the article/**Supplementary Material**. Further inquiries can be directed to the corresponding authors.
