# Supplementary material for: Diversity, expression, and structural modeling of sugar transporters in Anisakis simplex s. s. L3 and L4 larvae: an in vitro and in silico study
Source: Front Cell Infect Microbiol. 2025 Aug 20;15:1621051. doi: 10.3389/fcimb.2025.1621051 (PMC12405323; doi:10.3389/fcimb.2025.1621051)
Supplement: Supplementary file 1 [file DataSheet1.pdf]

Supplementary Material

1 Supplementary Figures and Tables

1.1 Supplementary Figures

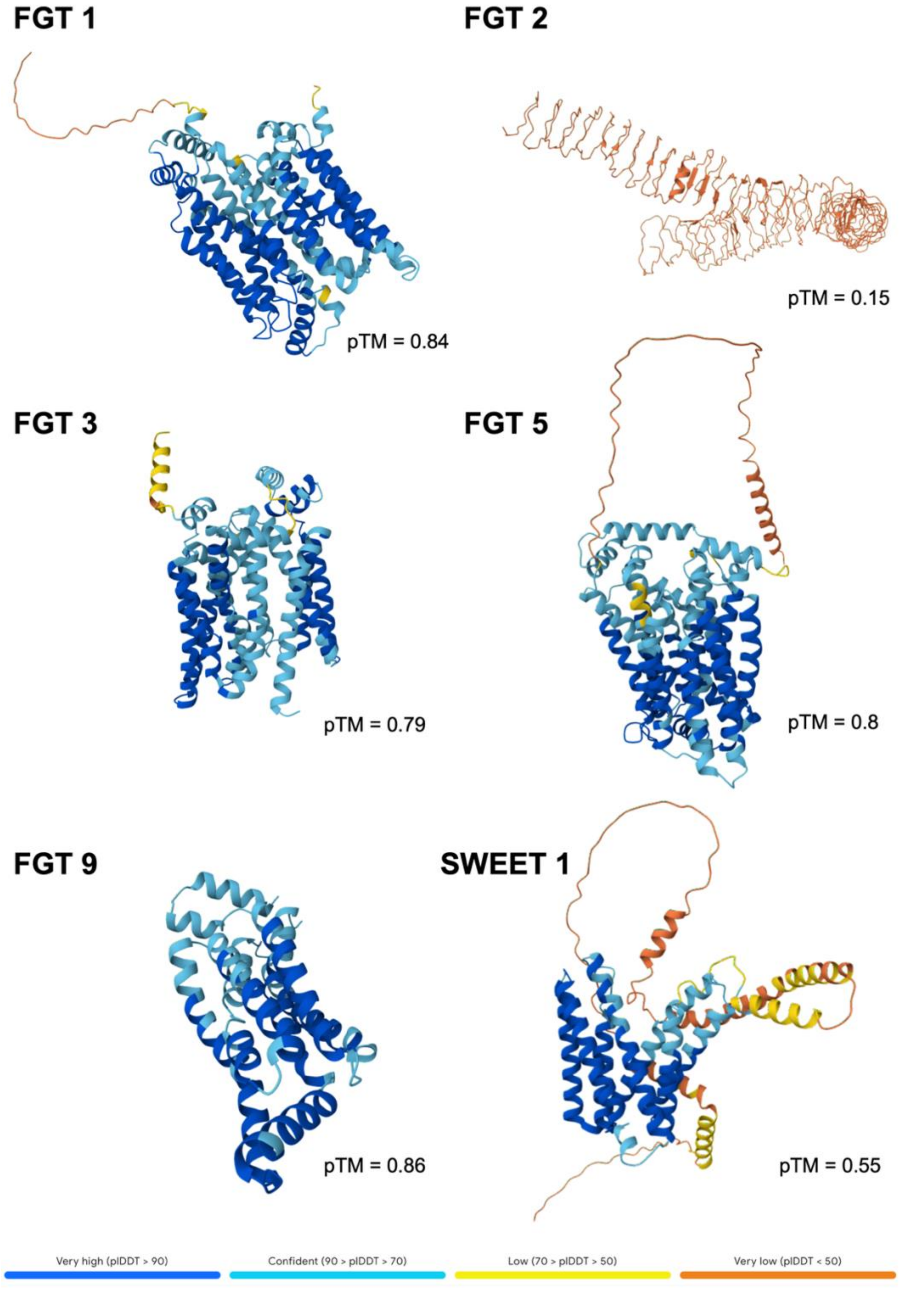

Supplementary Figure 1. Predicted tertiary structures of *Anisakis simplex* s. s. sugar transporters generated using AlphaFold 3 AI. Structural models are color-coded by per-residue confidence scores (pIDDT) on a scale from 0 to 100, where dark blue and blue indicates very high (>90), and high confidence (>70), respectively, yellow indicates low confidence (50-70), and orange denotes regions of very low confidence (<50). In addition, predicted template modeling (pTM) scores, which estimate the accuracy of the overall protein fold, were calculated. A pTM score above 0.5 indicates a high likelihood that the global structure closely approximates the native conformation.

Results: All predicted models displayed a typical transporter fold composed of multiple transmembrane helices, consistent with their classification within the Major Facilitator Superfamily (MFS). The majority of residues were modeled with high to very high local confidence (pLDDT > 70), particularly within the transmembrane domains, which are critical for substrate transport. Extracellular and intracellular loops showed slightly lower confidence scores, as expected due to their higher flexibility. The global accuracy of the predicted models was supported by the pTM scores, which ranged from 0.15 to 0.86 across the analyzed proteins, indicating a reliable approximation of the native fold. These values suggest that the predicted structures are suitable for comparative modeling and functional inference. Together, the structural features and confidence metrics validate the robustness of the AlphaFold 3 (Abramson et al., 2024) predictions for these parasite transporters and provide a solid foundation for future analyses.

References:

Abramson, J., Adler, J., Dunger, J., Evans, R., Green, T., Pritzel, A., et al. (2024). Accurate structure prediction of biomolecular interactions with AlphaFold 3. *Nature* 630, 493–500. doi: 10.1038/s41586-024-07487-w

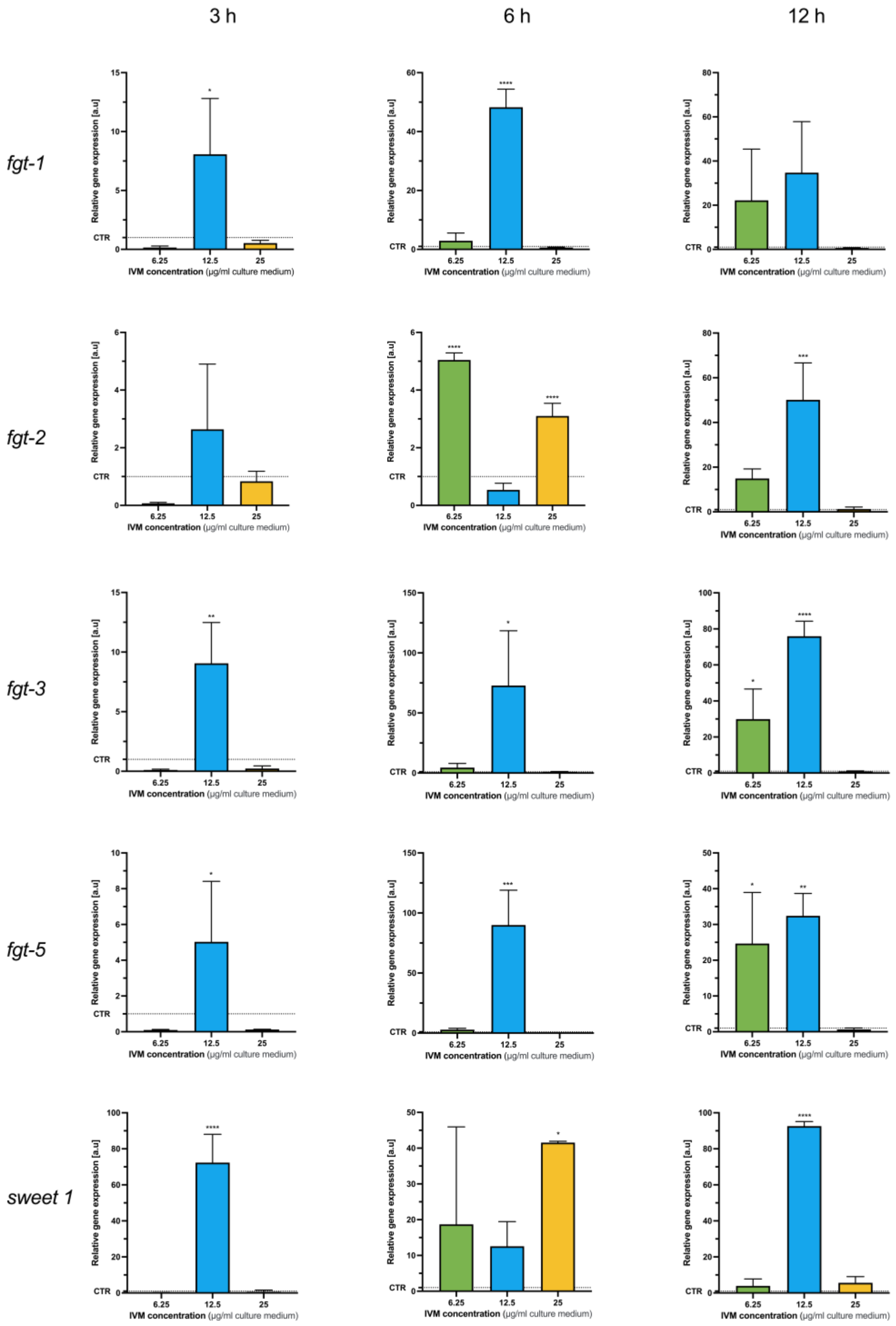

Supplementary Figure 2. The mRNA expression of *fgt-1, 2, 3, 5*, and *sweet-1* genes in L3 larvae of *A. simplex* s. s. exposed to different concentrations of ivermectin (6.25, 12.5, 25 µg/mL of culture medium) during 3h, 6h, and 12 h of an *in vitro* culture. Depicted values indicate the means of three replicates ± SD. The data were presented as the fold change in gene expression normalized to a reference gene *ef1-α* and relative to the untreated control (relative quantification RQ = 1). A one-way ANOVA analysis was performed and the differences between means were assessed by Tukey's

multiple comparisons test. P values were considered statistically significant, where 0.0332 (*), 0.0021 (**), 0.0002 (***), and <0.0001 (****). ns – non-significant results.

Results: Exposure to ivermectin led to significant, gene-specific changes in the mRNA levels of the glucose transporter family (*fgt*) and the sugar efflux transporter *sweet-1* in L3 larvae. In general, transcript levels increased with higher drug concentrations and longer exposure times (relative expression RQ = 1 in controls). For example, *fgt-1* was only slightly upregulated after 3 hours, but showed a pronounced, time-dependent induction (up to >10-fold above control) after 6 hours and 12 hours at 12.5 µg/mL. *Fgt-3* and *fgt-5* exhibited transient surges in expression: *fgt-3* peaked at 6 hours (~75-fold at the medium dose) before increasing further at 12 hours, while *fgt-5* was most strongly induced early (~100-fold at 6 hours) with a subsequent decline at 12 hours. *Fgt-2*, on the other hand, showed little change (maximum ~2–5-fold) at 6 hours, with expression observed at the highest IVM concentration. *Sweet-1* showed a response with a strong upregulation at 3 and 12 hours, with a decrease in the middle of the *in vitro* culture (6 hours). These results suggest that ivermectin induces a broad upregulation of sugar transporter genes in *A. simplex* s. s. L3, presumably as a compensatory mechanism to maintain energy homeostasis or stress adaptation under drug exposure. Furthermore, this supports the hypothesis that transporters can actively take up glucose from the environment even when pharyngeal uptake is inhibited by ivermectin treatment (Geary et al., 1993; Dube et al., 2022).

References:

Dube, F., Hinas, A., Roy, S., Martin, F., Åbrink, M., Svärd, S., et al. (2022). Ivermectin-induced gene expression changes in adult Parascaris univalens and Caenorhabditis elegans: a comparative approach to study anthelminthic metabolism and resistance in vitro. Parasit Vectors 15, 158. doi: 10.1186/s13071-022-05260-4

Geary, T. G., Sims, S. M., Thomas, E. M., Vanover, L., Davis, J. P., Winterrowd, C. A., et al. (1993). Haemonchus contortus: Ivermectin-Induced Paralysis of the Pharynx. Exp Parasitol 77, 88–96. doi: 10.1006/expr.1993.1064

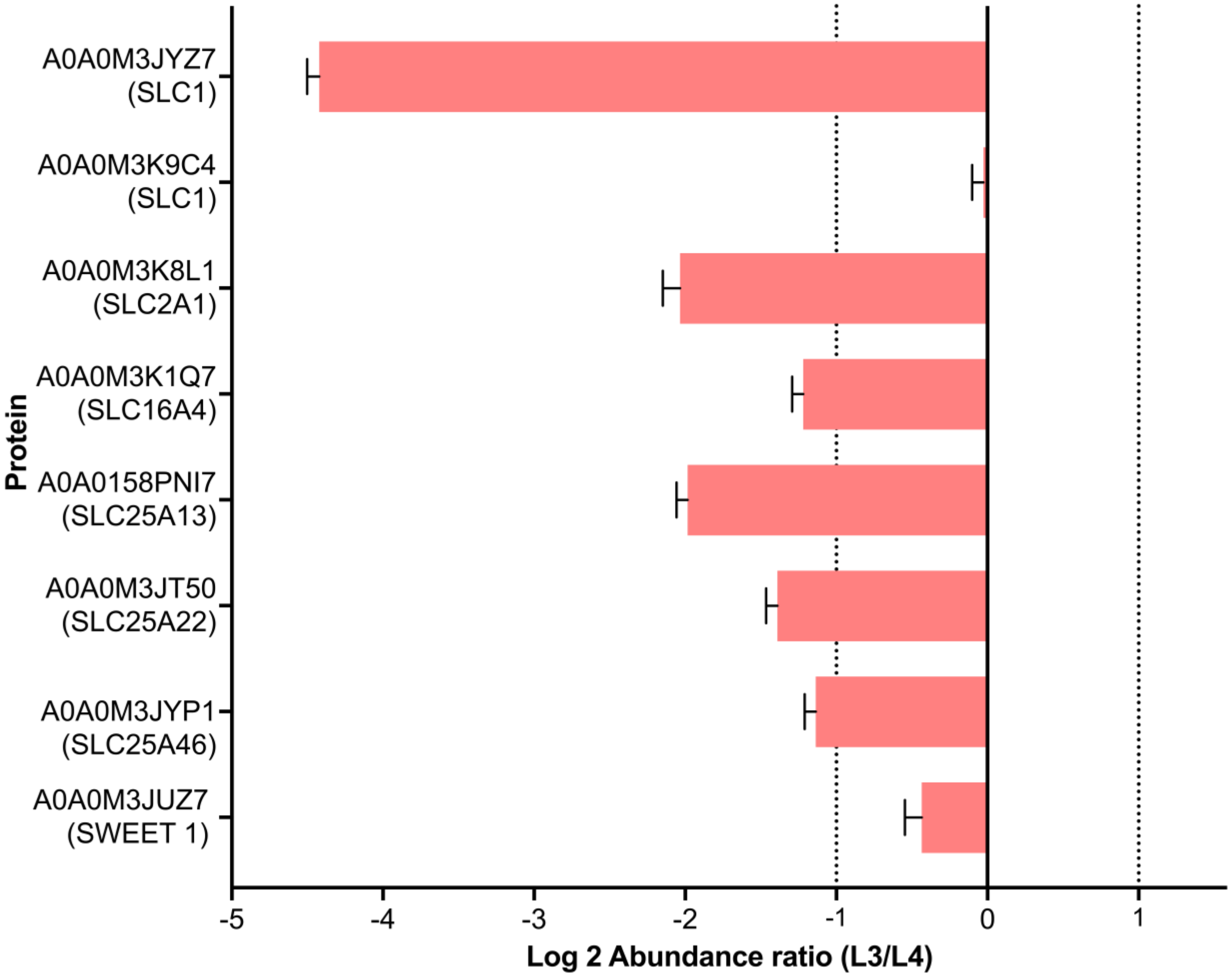

Supplementary Figure 3. The protein abundance of Solute Carrier (SLC) transporters and one of "Sugars Will Eventually Be Exported Transporters" (SWEET) determined between L3 and L4 stage larvae of *Anisakis simplex* by Stryiński et al. (2019, 2022) using LC-MS/MS.

Results: LC–MS/MS proteomic analysis revealed stage-specific abundance patterns for transporters of the SWEET and SLC family in *A. simplex* larvae. Nearly all identified transporter proteins were significantly more abundant in the parasitic L4 stage than in the L3 stage ($\log_2$ L3/L4 ratios of about –2 to –5, $p < 0.05$). This pronounced expression of nutrient transporters related to the L4 stage is consistent with the metabolic requirements of the feeding stage: as the L4 larvae reside in the gastrointestinal tract of the final host, they appear to upregulate carbohydrate import and metabolism to support active growth and energy consumption (consistent with proteomic data from Stryiński et al., 2019, 2022).

References:

Stryiński, R., Mateos, J., Carrera, M., Jastrzębski, J. P., Bogacka, I., and Łopieńska-Biernat, E. (2022). Tandem Mass Tagging (TMT) Reveals Tissue-Specific Proteome of L4 Larvae of Anisakis simplex s. s.: Enzymes of Energy and/or Carbohydrate Metabolism as Potential Drug Targets in Anisakiasis. *Int J Mol Sci* 23, 4336. doi: 10.3390/ijms23084336

Stryiński, R., Mateos, J., Pascual, S., González, Á. F., Gallardo, J. M., Łopieńska-Biernat, E., et al. (2019). Proteome profiling of L3 and L4 Anisakis simplex development stages by TMT-based quantitative proteomics. *J Proteomics* 201, 1–11. doi: 10.1016/j.jprot.2019.04.006

1.2 Supplementary Tables

Supplementary Table 1. The list of primers used for real-time PCR.

Name	No. accession GenBank	Forward sequence: (5' to 3')	Reverse sequence: (5' to 3')
fgt-1	MF069077	TCCCTAGAATGTGCACGCTG	AATGCGGCCGTCGTATTTTG
fgt-2	MG557622	CATGAGAGCGGAGTGTTTGA	ATGAATTTCGCGACCTTTTG
fgt-3	MG557623	TCCGGCAGTGTTTTCTAACC	TCGATGTCCGCAAATATGAA
fgt-5	MG210725	AGGCGAGAGAGAAGGAGGTT	ATTATCGTTGGCTGCACGGA
fgt-9	MG557624	CGTTTGATATTTGGTCAGGCTA	GGTCTACTGCTCTGTGCTGG
sweet-1	MG210740	GCACCATTAAAAATATCACCGTAA	TTCGAAAACCCGATGAATCT
ef-1 α	KP326558	TCCTCAAGCGTTGTTATCTG	AGTTTTGCCACTAGCGGTT

Supplementary Table 2. The characterization of glucose transporter sequence exons in *Anisakis simplex, Toxocara canis, Caenorhabditis elegans* and humans. The visualization of the genomic organization of selected facilitative glucose transporters is shown in Figure 1.

	FGT1 A.simplex			FGT1 H.sapiens			FGT1 T.canis			FGT1 C.elegans		
	**MF069077**			**NM_006516**			**KHN73404**			**NM_061580**		
	**start**	**end**	**length**	**start**	**end**	**length**	**start**	**end**	**length**	**start**	**end**	**length**
**CDS**	1	4 244	1 557	1	31 611	1 479	1	8 694	1 461	1	5 120	1 479
**exon 1**	1	105	105	1	18	18	1	149	149	1	63	63
**exon 2**	327	428	102	15 331	15 426	96	618	742	125	359	457	99
**exon 3**	735	817	83	27 446	27 606	161	1 278	1 432	155	1 139	1 218	80
**exon 4**	975	1 180	206	27 786	28 026	241	2 315	2 526	212	1 315	1 520	206
**exon 5**	1 488	1 658	171	28 617	28 779	163	4 881	5 010	130	1 983	2 217	235
**exon 6**	2 064	2 200	137	28 872	29 059	188	5 555	5 682	128	2 502	2 658	157
**exon 7**	2 677	2 949	273	29 338	29 442	105	7 144	7 374	231	3 785	4 093	309
**exon 8**	3 141	3 233	93	29 619	29 720	102	7 953	8 094	142	4 172	4 363	192
**exon 9**	3 450	3 537	88	30 844	31 047	204	8 506	8 694	189	4 983	5 120	138
**exon 10**	3 644	3 771	128	31 411	31 611	201	-	-	-	-	-	-
**exon 11**	4 074	4 244	171	-	-	-	-	-	-	-	-	-

	FGT3 A.simplex			FGT3 H.sapiens			FGT3 T.canis			FGT3 C.elegans		
	**MG557623**			**NM_006931**			**KHN77673**			**NM_067319**		
	**start**	**end**	**length**	**start**	**end**	**length**	**start**	**end**	**length**	**start**	**end**	**length**
**CDS**	1	4 700	1 313	1	14 622	1 491	1	10 290	1 512	1	3 010	1 518
**exon 1**	1	117	117	1	15	15	1	75	75	1	116	116
**exon 2**	843	997	155	2 133	2 225	93	941	1 031	91	163	229	67
**exon 3**	1 384	1 513	130	2 888	3 048	161	2 041	2 157	117	914	1 365	452
**exon 4**	1 691	1 816	126	4 550	4 790	241	2 612	2 766	155	1 421	1 678	258
**exon 5**	2 159	2 307	149	5 393	5 555	163	3 279	3 408	130	1 733	2 117	385
**exon 6**	2 888	2 965	78	6 164	6 351	188	4 104	4 229	126	2 771	3 010	240
**exon 7**	3 372	3 592	221	10 087	10 191	105	5 136	5 284	149	-	-	-
**exon 8**	4 011	4 127	117	11 524	11 625	102	6 613	6 690	78	-	-	-
**exon 9**	4 481	4 700	220	13 011	13 214	204	7 465	7 685	221	-	-	-
**exon 10**	-	-	-	14 404	14 622	219	8 628	8 744	117	-	-	-
**exon 11**	-	-	-	-	-	-	10 038	10 290	253	-	-	-

	FGT5 A.simplex			FGT5 H.sapiens			FGT5 T.canis			FGT5 C.elegans		
	**MG210725**			**NM_003039**			**KHN84357**			**NM_061580**		
	**start**	**end**	**length**	**start**	**end**	**length**	**start**	**end**	**length**	**start**	**end**	**length**
**CDS**	1	3 634	1 644	1	31 951	1 506	1	10 364	1 629	1	5 120	1 479
**exon 1**	1	126	126	1	33	33	1	102	102	1	63	63
**exon 2**	383	469	87	11 287	11 385	99	846	941	96	359	457	99
**exon 3**	677	778	102	11 929	12 089	161	2 392	2 493	102	1 139	1 218	80
**exon 4**	1 023	1 305	283	21 803	21 927	125	3 013	3 089	77	1 315	1 520	206
**exon 5**	1 414	1 584	171	27 600	27 752	153	3 377	3 582	206	1 983	2 217	235
**exon 6**	1 876	2 060	185	29 348	29 473	126	4 369	4 539	171	2 502	2 658	157
**exon 7**	2 158	2 385	228	29 550	29 737	188	5 286	5 470	185	3 785	4 093	309
**exon 8**	2 532	2 630	99	29 875	29 985	111	5 971	6 198	228	4 172	4 363	192
**exon 9**	3 043	3 127	85	30 608	30 709	102	8 153	8 251	99	4 983	5 120	138
**exon 10**	3 206	3 333	128	31 031	31 106	76	8 689	8 773	85	-	-	-
**exon 11**	3 485	3 634	150	31 513	31 640	128	9 270	9 397	128	-	-	-
**exon 12**	-	-	-	31 748	31 951	204	10 215	10 364	150	-	-	-
